# Southern leaf blight disease severity is correlated with decreased maize leaf epiphytic bacterial species richness and the phyllosphere bacterial diversity decline is enhanced by nitrogen fertilization

**DOI:** 10.3389/fpls.2014.00403

**Published:** 2014-08-15

**Authors:** Heather C. Manching, Peter J. Balint-Kurti, Ann E. Stapleton

**Affiliations:** ^1^Department of Biology and Marine Biology, University of North Carolina WilmingtonWilmington, NC, USA; ^2^USDA-ARS and Department of Plant Pathology, North Carolina State UniversityRaleigh, NC, USA

**Keywords:** phyllosphere, southern leaf blight, abiotic stress, fertilizer, nitrogen, epiphyte, ribosomal diversity, B73

## Abstract

Plant leaves are inhabited by a diverse group of microorganisms that are important contributors to optimal growth. Biotic and abiotic effects on plant growth are usually studied in controlled settings examining response to variation in single factors and in field settings with large numbers of variables. Multi-factor experiments with combinations of stresses bridge this gap, increasing our understanding of the genotype-environment-phenotype functional map for the host plant and the affiliated epiphytic community. The maize inbred B73 was exposed to single and combination abiotic and the biotic stress treatments: low nitrogen fertilizer and high levels of infection with southern leaf blight (causal agent *Cochliobolus heterostrophus*). Microbial epiphyte samples were collected at the vegetative early-season phase and species composition was determined using 16S ribosomal intergenic spacer analysis. Plant traits and level of southern leaf blight disease were measured late-season. Bacterial diversity was different among stress treatment groups (*P* < 0.001). Lower species richness—alpha diversity—was correlated with increased severity of southern leaf blight disease when disease pressure was high. Nitrogen fertilization intensified the decline in bacterial alpha diversity. While no single bacterial ribotype was consistently associated with disease severity, small sets of ribotypes were good predictors of disease levels. Difference in leaf bacterial-epiphyte diversity early in the season were correlated with plant disease severity, supporting further tests of microbial epiphyte-disease correlations for use in predicting disease progression.

## Introduction

Corn, *Zea mays* L., is one of the most widely grown crops worldwide and is especially important economically (http://www.nass.usda.gov). Management of this crop has changed substantially in the last few decades, in concert with development of improved genotypes (Ciampitti and Vyn, 2012; Grassini and Cassman, 2012). Nitrogen fertilizer application is a key component for yield in modern maize genotypes, but the cost and effects of over-application have generated pressure to reduce use (Cui et al., 2013). Nitrogenous fertilizer use is expected to increase at a slower rate in well-resourced regions, while increasing in Asia and much of Africa (Rengel, 2013). A second important limit on maize yield is fungal infection. Southern Leaf Blight (SLB) is caused by the fungus *Cochliobolus heterostrophus (Drechs.) Drechs*. (*anamorph = Bipolaris maydis (Nisikado) Shoemaker; synonym = Helminthosporium maydis Nisikado*) SLB caused a severe epidemic throughout the US in 1970 due to the widespread presence of susceptibility alleles in production hybrid genotypes (Ullstrup, 1972). It is now mainly a problem in tropical and sub-tropical maize growing areas in the southeastern US and parts of Asia and Africa. Spores from this fungus land on the leaf surface, germinate, and penetrate either directly through the stomata or the leaf cuticle and epidermis (Jennings and Ullstrup, 1957). This pathogen spreads from leaf litter and can produce windborne spores within days (Horwitz et al., 2013). As fungal infection progresses the leaf develops characteristic lesions and resource allocation to reproductive tissues can suffer (Byrnes, 1989). The disease is normally controlled by incorporation of resistant alleles (Balint-Kurti et al., 2007; Kump et al., 2011); resistance, however, is labor-intensive to score and can only be measured late in the growing season.

A wide range of bacteria and other organisms inhabit the outer surface (epiphytes) and inner tissues (endophytes) of all plants (Newton et al., 2010). Leaf surfaces provide an estimated foliar area of 10^8^ km^2^ (Lindow and Brandl, 2003) that could be colonized. Epiphytes inhabiting leaf surfaces (the phyllosphere) are exposed to conditions including drought, high UV exposure, varying temperatures, and low nutrient availability (Lindow and Brandl, 2003). In response, many epiphytes have developed survival strategies and can potentially alter their environment to better suit them (Newton et al., 2010). Phyllosphere microbiota could contribute to the overall health of a plant species through both promotion of growth and surface protection against pathogens (Lindow and Brandl, 2003). Since both the host plant and the fungal pathogen can have associated bacteria, to fully understand the determinants of pathogen spread we need to understand the dynamics of the bacterial affiliates (Kemen, 2014). Though there is relatively little information on the time scales and plasticity of these multi-way interactions, there is one report of increased bacterial diversity in the presence of a beneficial fungal endophtye (Yang et al., 2013). Different rice varieties are capable of harboring different epiphytic communities on their leaves, which potentially have the ability to act as antagonists of *Rhizoctonia solani*, the sheath blight pathogen of rice (De Costa et al., 2006a,b). In maize, growth-promoting and antifungal-producing bacteria have been shown to have inhibitory effects on SLB disease (Huang et al., 2010; Ye et al., 2012).

Both the number of different microbial species, the alpha diversity, and the arrangement of species across experimental groups, the beta diversity are relevant when examining phyllosphere community development, structure and function (Meyer and Leveau, 2012). Antagonistic interactions with pathogens could be due to either stochastic or deterministic processes (Caruso et al., 2012) such as competition for nutrients, excretion of toxins, and shared metabolic pathways. For use of microbial community structure information for better disease suppression, the stability across spatial scales (beta diversity) and the number of different sets of health-associated communities are important parameters to examine. The presence of multiple species in a single environment can allow for competitive exclusion of certain species (Meyer and Leveau, 2012). Balint-Kurti et al. (2010) proposed that the presence of certain species of bacteria on corn leaves could increase resistance to *C. heterostrophus* infection. In their study, the more disease-susceptible B73 genotype leaves hosted fewer bacterial types with significantly more variation of the bacterial community. A second study supported a model where disease-facilitating bacteria colonize B73, though bacterial diversity was not assayed directly (Balint-Kurti et al., 2011). It is not yet clear whether community structure and disease resistance are causally related or independent processes–disease resistance could be due to loss of facilitating species and/or to high beta diversity and competitive community structure.

Results of previous studies on SLB and leaf bacterial populations suggested that pathogen-suppressive bacterial populations might be important, though since most leaf bacteria are as yet uncultured the evidence for suppressive bacteria is indirect. In the Balint-Kurti et al. (2011) study, antibiotic was applied to maize leaves from SLB-resistant and SLB-susceptible inbreds and SLB disease severity was reduced in the susceptible inbred; bacterial beta diversity in these inbreds was negatively correlated with the SLB disease severity in a previous study (Balint-Kurti et al., 2010), suggesting a model with bacterial pathogen-suppressive bacteria joining the leaf community after antibiotic disruption. If specific suppressive pathogen-attacking bacterial species colonize leaves, then factors that affect growth of the plant should not affect overall bacterial diversity patterns, but instead should affect specific bacterial species or sets of species.

The microhabitat that is present on maize leaves has not been studied extensively in single or combined stress environments. There is a positive correlation between SLB severity, fertilizer levels and irrigation in maize (Bekele, 1983). However, the relationship between pathogen effect and bacterial diversity has not been examined under managed environmental stress. Here we examined how microbial communities, plant traits and disease severity changed when exposed to combinations of abiotic and biotic stresses. It is expected that the overall growth success of a plant would decrease as more stress is applied, with combinations of multiple stresses having more impact. Since SLB disease is typically more severe in the presence of fertilizer, bacterial epiphyte beta diversity would be predicted by the Balint-Kurti et al. (2011) model to decrease under these conditions; abundance of disease-suppressive ribotypes, if important, would be expected to decrease as well.

## Materials and methods

### Experimental design

Seed of the B73 maize inbred was provided by the Maize genetics cooperation stock center (http://maizecoop.cropsci.uiuc.edu/) and increased under standard nursery conditions at the North Carolina Central Crops Research Station. The experimental plots were planted April 16, 2012 at the North Carolina Central Crops Research Station (35.66979°, −78.4926°). Weather data for this site is available from http://www.nc-climate.ncsu.edu/cronos/index.php?station=CLAY. There were a total of 4 treatment groups consisting of combinations of the following factors: no nitrogen fertilizer (unfertilized plots) or normal nitrogen fertilization (fertilized plots), and inoculation with Southern Leaf Blight (pathogen-inoculated plots) or fungicide spray to reduce SLB levels (fungicide plots). The fertilized fungicide-treated blot corresponds to normal corn nursery growth conditions for this site. This experiment was part of a larger field experiment, with the ten B73 genotype plots randomly placed among plots from different genotypes and the same plot design for genotypes within each field treatment block (plot map in Supplemental File S1) (Manching, 2013). Normal fertilizer application for this site was preplant of 86.8 kg ha^−1^ 10-10-30 (21.5 kg of nitrogen), side-dress of 112 l ha^−1^ of 24S liquid nitrogen yielding 13.39 kg of nitrogen and then top-dress with 476 l ha^−1^ of 24S liquid nitrogen for another 56.9 kg of nitrogen. Total nitrogen applied to the fertilizer block was 37.1 kg ha^−1^. Treatment blocks without fertilizer had no preplant or side-dress application. The field was thinned 4 weeks after planting by removing plants that were growing less than 8 inches apart.

### Inoculation with southern leaf blight

Plants were inoculated with Southern Leaf Blight (SLB) on May 24, 2012 at the four-to-six leaf growth stage (V4). Inoculation was performed by placing 20–30 grains of *C. heterostrophus* race O, isolate 2–16 Bm sorghum grain culture in the leaf whorl of each plant (Balint-Kurti and Carson, 2006). The fungicide Headline (BASF Co.) was applied on plants that were not inoculated at a rate of 9 ounces per acre for the first two applications on June 8 and July 6, 2012 and the fungicide TEBU 3.6 (Amtide LLC, Irvine, CA) was applied at a rate of 6 ounces per acre for the final application on July 26, 2012, in order to reduce spread of pathogen outside the designated treatment groups.

### Sampling of bacterial populations

Bacterial population samples were collected on June 15, 2012 at the V7 stage. Samples were collected by rubbing 12.5 mm diameter filter paper discs on each side of one leaf per plant using sterile technique. Four plants were sampled for each entry (adjacent planted set) of B73 plants, for a total of 40 samples per treatment group. Filter paper samples were immediately stored frozen in sterile bags (Whirl-Pak, Nasco, Inc.) until DNA was extracted. This is an equal effort sampling design, which allows straightforward comparisons across samples without sample weighting.

Bacterial DNA was extracted from the filter discs using the MoBIO UltraPure DNA Extraction Kit. A nested automated ribosomal intergenic spacer analysis (Lear and Lewis, 2009) was used to amplify DNA samples for analysis using Phire DNA Polymerase and buffer (Thermo Scientific Inc.), universal bacterial primers (Lear and Lewis, 2009) ITSF and LD for the first run and ITSReub and SD for the second run, and 10 mM dNTPs. The first run samples were cycled once at 95°C for 30 s, 35 times at 95°C for 30 s, 61.5°C for 30 s, and 72°C for 1 min, 30 s, and finally once at 72°C for 10 min (Lear and Lewis, 2009). One microliter of the first run sample was used in the second run following the same cycle as described above. Final bacterial samples were combined with a solution of HiDi and ROX500 size standard (ABI, Foster City, CA) and heated to 94°C for 10 min to separate fragments. Fingerprints of fragment lengths were analyzed using an ABI 3130 Genetic Analyzer (ABI, Foster City, CA).

### Measurement of plant and cob traits

Plant traits including cob diameter, seed weight, and plant height were measured for each plant in the field. All plant data are provided in Supplemental Data File S2. Plant height measurements were taken by measuring from the base of the plant to the top of the tassel, if present, before harvest. Cob diameter was measured on the base of the dried ears obtained after harvest. For seed weight, 20 seeds, if present, were removed from each cob and weighed to the nearest hundredth of a gram. If the cob did not have enough seeds, it was not included in the seed weight dataset.

SLB disease severity was scored on July 17, 2012. Scores are obtained based on a scale from 1 to 9, with 9 representing no evidence of Southern Leaf Blight and 1 representing a plant that is completely brown due to presence of SLB (Balint-Kurti and Carson, 2006; Kump et al., 2011). Every plant within an entry was categorized together and one score was given for each entry as a whole. Entries that could not be scored due to the inability to determine if leaf lesions were caused by SLB were not included in the analysis.

### Statistical analysis

DNA fragment sizes were binned using the histogram bin width optimization method as previously described by Shimazaki and Shinomoto (Shimazaki and Shinomoto, 2007). Each bin was categorized by an operational taxonomic unit (OTU) number. A microbial community structure profile was created for all factors using the RiboSort (Scallan et al., 2008) package in R (R Development Core Team, 2005), with only peaks that were consistently present—in both amplification reactions from the sample DNA—being included in the abundance calculation. All sample OTU counts are included in Supplemental File S3, S4. Differences in alpha and beta diversity were analyzed using the ADONIS, MDS, and BETADISPER functions located in the Vegan package in R, and significance tested using permdisp2 and permanova (Anderson, 2001, 2004). Transformation, standardization and dissimilarity methods were systematically varied to determine the robustness of the diversity comparisons (Supplemental File S1). Contribution of individual operational taxonomic units to environment differences was calculated using the SIMPER function in the PAST program (Hammer et al., 2001) using default parameters; the PAST program was also used to generate the phyllosphere partial correlation plots with 95% ellipsoids and to calculate the Chao1 alpha diversity values for each sample. Plant trait effects and correlations between the Chao1 diversity and the disease severity score were analyzed using SAS JMP v11Pro. Plant traits and pathogen scores are provided in Supplemental File S2 and bacterial community data in Supplemental Files S3–S5.

Multivariate regression trees (MRT) analysis was implemented using the MVpart v1.6-1 package for R Development Core Team (2005). MRT was developed as a statistical tool to explore relationships between environments and the multiple species that inhabit them (De'ath and Team, 2012). This analysis uses repeated splitting to form clusters from the data, with each cluster representing a simple change or rule based on environmental factors. This method of analysis was used to generate a multivariate perspective on the relationships between multiple phenotypes and the stress treatments. Separate analyses were conducted, one for all of the traits including the Simpson diversity in all four treatment environments and a second for all of the plant traits and the alpha and beta diversity (calculated with the R package vegetarian), shown in Supplemental File S1. Missing data were imputed with the R package MI. For comparison, trait values computed in the original measurement scale are included as bar graphs on the branches of the multivariate split tree.

## Results

### Phyllosphere diversity and pathogen levels

**Figure 1:**
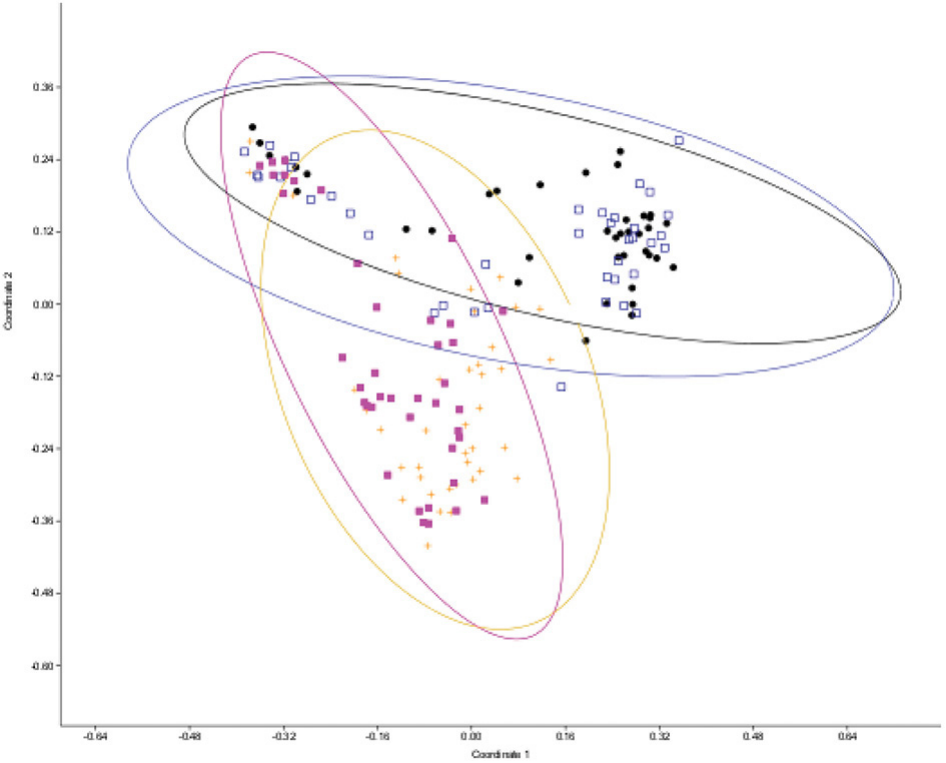
**Principal coordinates plot of leaf epiphyte microbial diversity as measured by ribosomal spacer length differences**. Distance from centroid and 95% confidence ellipses are shown for each treatment level. Each point represents a microbial sample from one plant. Black squares, unfertilized, fungicide-treated plots; hollow blue squares, fertilized fungicide-treated plots; pink squares, fertilized and pathogen-inoculated plots; orange plus symbols, unfertilized and pathogen-inoculated plots.

In order to examine the effect of abiotic and biotic stress on phyllosphere diversity, bacterial communities were analyzed for differences among the treatment groups. The treatments that were used in analysis included the following: unfertilized with SLB pathogen infection controlled by fungicide spray (referred to as fungicide), fertilized and inoculated with SLB (referred to as pathogen-inoculated), unfertilized and pathogen-inoculated, and control plots (fertilized, treated with fungicide to control SLB). The abundance and ribotypes of the bacterial community were compiled and community diversity values were calculated for each treatment group. Groups that had the same pathogen treatment (pathogen-inoculated vs. fungicide sprayed) cluster together by principal coordinates projections, as can be seen by comparison of the orange and pink relative to blue and black symbols in Figure 1. There was a significant difference observed in diversity between the four treatments when analyzed by permanova (*p* < 0.001 for overall model, with significant *P*-values in all tested combinations of distance measure, transformation, and standardization parameters, Supplemental File S1 Table S1).

We compared the bacterial alpha diversity, summarized as the Chao1 index, to late-season disease severity. As expected, disease was more severe in pathogen-inoculated plots, with disease scores significantly lower in pathogen-inoculated plots (Table 1). As the Chao1 alpha diversity index measure exhibited different multivariate distributions in the pathogen-inoculated plots relative to the fungicide-treated plots (Supplemental File S1 Table S2), we could only compare the index within pathogen levels. Leaf bacterial alpha diversity was lower in pathogen-inoculated plots, with significantly lower diversity in the pathogen-inoculated fertilized samples than the pathogen-inoculated unfertilized samples (Table 1). The correlation between SLB disease severity score and Chao1 alpha diversity is high and positive in every case except for fungicide-treated fertilized plots (Table 1).

**Table 1:** **Microbial diversity and disease severity**.

		Mean Chao1 alpha diversity^*^	**Mean leaf SLB severity score^§^**	**Correlation between Chao1 diversity and disease score^¶^**
Pathogen-inoculated	Fertilized	52.1^A^	5.5^A^ (more disease)	0.454 (0.728, *P* < 0.0001)
Pathogen-inoculated	Unfertilized	69.7^B^	6.1^A^	0.206 (0.445, *P* < 0.0001)
Fungicide	Fertilized	76.7^a^	7.3^B^	−0.101 (−0.203, *P* = 0.0016)
Fungicide	Unfertilized	81.0^a^	8.0^B^ (less disease)	0.151 (0.479, *P* < 0.0001)

* *Within pathogen-inoculated samples pairwise Wilcoxon P = 0.0115, within fungicide samples Wilcoxon P = 0.64; different letters indicate significantly different comparisons with A significantly different than B and the two a labels not significantly different from each other*.

§ *P-values from ordinal logistic fit are given in Supplemental File S1, Table S6*.

¶ *correlation with missing data imputed is given in parentheses, along with with P-value for Spearman rho test of correlation significance using the imputed dataset*.

Beta diversity was measured as average distance to the multivariate centroid, and was pairwise significant under some combinations of distance measure, transformation, and standardization methods when the treatment blocks were compared (Supplemental File S1 Table S1), though the overall model was not significant by permutation. Within each block, the 10 plot replicates of four plants were tested for beta diversity differences, and the fungicide-treated blocks had significantly higher dispersion (Table 2).

**Table 2:** **Microbial beta diversity within treatment blocks**.

**Stress treatment type**	**Average deviation**^§^	**P(permutation)^¶^**
Pathogen-inoculated fertilized	3.5	0.79
Pathogen-inoculated unfertilized	3.6	0.39
Fungicide fertilized	5.8	0.038^*^
Fungicide unfertilized	6.8	0.02^*^

§ *Average devation from the mean of the distance to multivariate centroid for the 10 replicates within each block*.

¶ *P-value for overall model from permdisp2 analysis of the 10 blocks of four replicate plants*.

* *P< 0.05*.

### Specific ribotype contributions to communities

**Figure 2:**
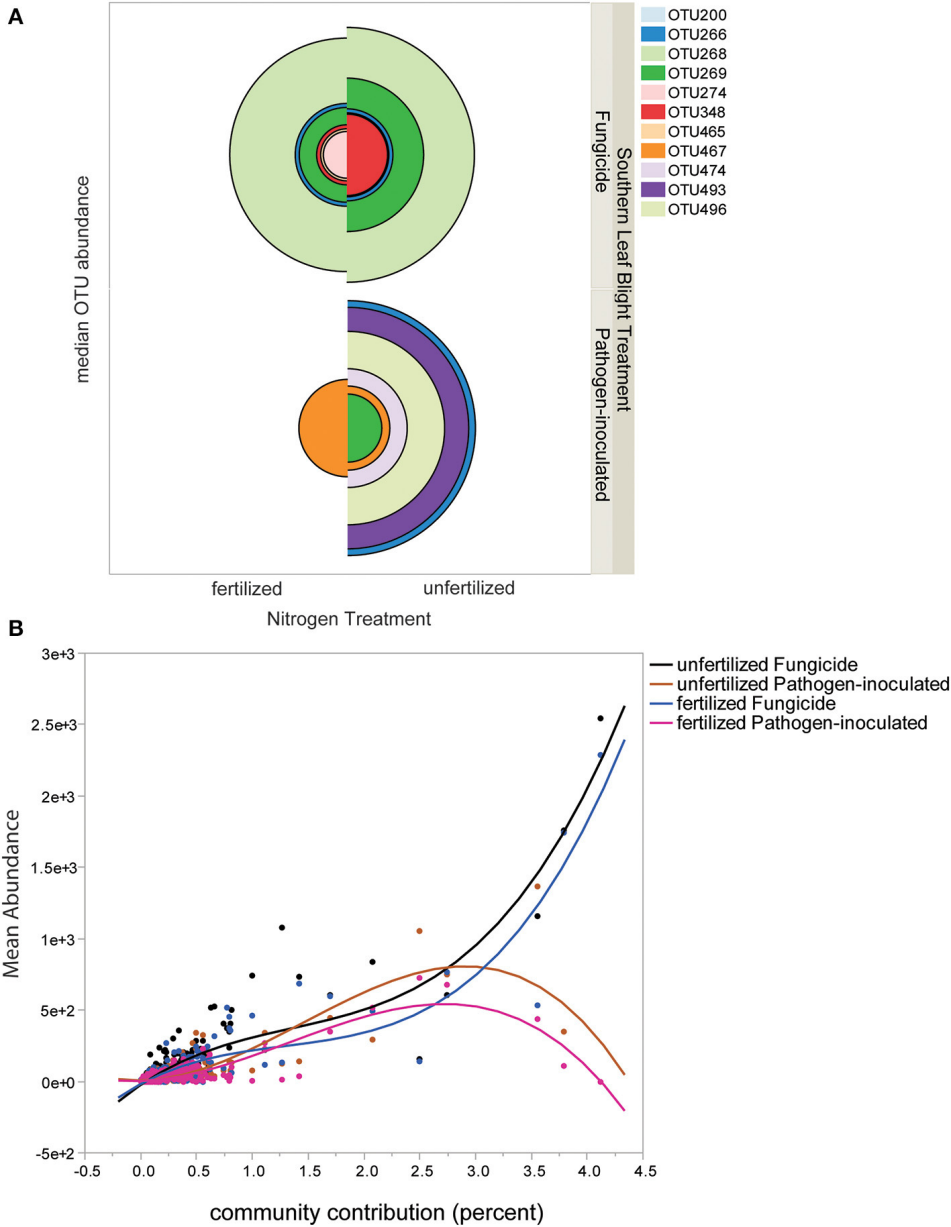
**Distribution of individual ribotypes in each treatment level. (A)** Twelve OTU contributing more than two percent to treatment level differences by SIMPER analysis are shown, with the size of the pie chart segment indicating the median relative abundance of the OTU. Each OTU is identified by the ribotype size number in parentheses, and the size of the quadrant for each is the median abundance of the ribotype in that sample. **(B)** Plot of OTU abundances ordered by SIMPER Bray-Curtis percent of contribution to community. A cubic fit line to each treatment group is shown. The line fit statistics are: Mean Relative Abundance unfertilized Fungicide: RMSE = 61.71, *R*^2^ = 0.84. Mean Relative Abundance unfertilized Pathogen-inoculated RMSE = 45.62, *R*^2^ = 0.69. Mean Relative Abundance fertilized Fungicide RMSE = 52.63, *R*^2^ = 0.83. Mean Relative Abundance fertilized Pathogen-inoculated RMSE = 28.53, *R*^2^ = 0.73.

The lists of ribotypes in each set of samples were examined for individual operational taxonomic units (OTU) that contributed most to treatment differences. Eleven OTU contributed to greater than 1% of the differences between communities in both Bray-Curtis and Euclidian measure SIMPER analyses. The median relative abundance of the eleven largest-contributing species is visualized in Figure 2A. The pie chart sections indicate that in some cases there were a small set of SIMPER-identified important ribotypes (and many more with very small contributions), such as OTU467 in the fertilized pathogen-inoculated treatment; OTU467 is the largest half-circle in this quarter of the graph and thus has the largest contribution. In other cases such as the unfertilized pathogen-inoculated there are six OTU that contribute strongly to the differences, as illustrated by the six larger-sized half-circles (Figure 2A, lower right quadrant). Different ribotypes can be observed in pathogen-inoculated and fungicide-sprayed samples; for example, OTU268 is present only in fungicide-sprayed phyllosphere samples. In addition to the median view of important ribotypes, we also examined the distribution of ribotypes, by plotting the relative abundance by the community contribution importance (Figure 2B). Both pathogen-inoculated treatments had a more even distribution of ribotypes, with important ribotypes showing relatively low abundances, while the fungicide-treated samples contained ribotypes with high relative abundance and high importance (Figure 2B). Fungicide-treated plants have more leaf bacterial diversity and the most abundant ribotypes are present in both fertilized and unfertilized plots, in contrast to pathogen-inoculated plants, which have less diversity in fertilized plots but more OTU with high abundance. Thus, it is not just a gradient of species richness, but specific species sets that that are correlated with severe disease (e.g., OTU496, 474, 493) or correlated with little disease (OTU269).

### Multivariate regression tree analysis

**Figure 3:**
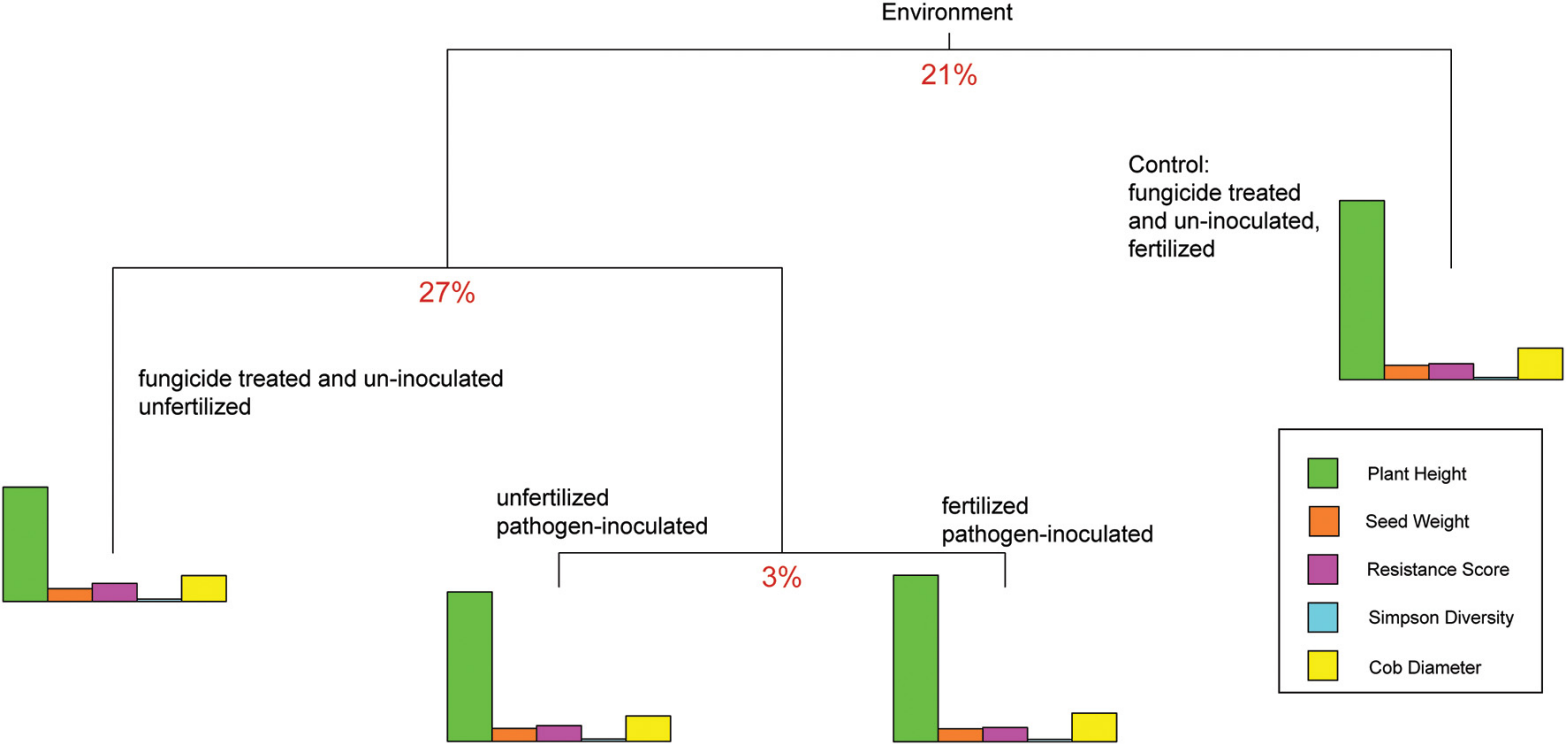
**Multivariate partition results for plant traits**. The partition explaining the most multivariate variance is at the top of the branch diagram, with each subsequent split dividing the remaining variation. The splitting variables are grouped together to start (as “environment”) then split by stress treatment combination. For reference, the mean trait values (rather than the multi-dimensional matrices) are plotted as bar graphs for each split. The relative contribution of each trait down each branch can be evaluated by comparing bar heights.

To examine the relative effects of the different abiotic and biotic treatments on the traits, the response variables of phyllosphere bacterial Simpson diversity and the plant traits plant height, seed weight, SLB severity, and cob diameter were used for an analysis which included the following treatments: (1) fertilized and treated with fungicide, (2) unfertilized and treated with fungicide, (3) pathogen-inoculated and unfertilized, and (4) fertilized and pathogen-inoculated. With treatment set as the predicting variable, data were split into three groups, which explained 51% of the phenotypic variance. The first split, which explains 21% variance, resulted in the separation of the fertilized fungicide treatment group (Figure 3) from all others. The second split, which explained 27% variance, resulted in the separation of the fungicide-treated unfertilized plants from all pathogen-inoculated plants (3%). The analysis was repeated using separate alpha and beta diversity values instead of the overall Simpson diversity index; this generated the same order of splits but slightly different percent variance to each split (23, 27, and 4%). The three high-stress treatments split from unstressed plot data. Pathogen-inoculated plants grouped together in the mvpart analysis until the final split.

### Plant traits

Plant traits were measured late in the season. Plant height and seed weight showed the expected decreases in stress treatment blocks (Supplemental Tables S4–S6), though with non-addititivity in some combinations of stress treatments. In particular, pathogen-inoculated unfertilized plants were taller than pathogen-inoculated fertilized plants (Supplemental Table S4), which was not consistent with an additive restraint on growth from biotic and abiotic stress. The taller unfertilized pathogen-inoculated plants had a larger set of ribotypes of high-relative-abundance bacteria (Figure 2), suggesting the nitrogen fertilizer may limit pathogen growth more severely than bacterial colonization.

## Discussion

We studied how microbial communities, plant traits and disease severity changed when exposed to combinations of abiotic and biotic stress in the field. The association of increased nitrogen availability with increased disease levels has been noted previously in maize and other crops (Shaner and Finney, 1977; Bekele, 1983; Caldwell et al., 2002) and was confirmed in our study. Our experiment was designed to compare treatments within one soil type in a setting with the best possible control of field variation. Since SLB spores spread rapidly and are pervasive in this region of the USA, we used fungicide to control infection, and compared fungicide-treated plots to pathogen-inoculated plots. Fungicide treatment for SLB control has been previously used at this site and generates substantial differences in disease severity between inoculated and fungicide-treated plots (Santa-Cruz et al., 2014); we also saw a consistent difference in disease severity in our study (Table 1). Since we only compared the inoculated plots to fungicide-treated plots, we cannot necessarily extrapolate our results to wind-inoculated maize plants at this location. Uncontrolled inoculation is more variable in time and space. In a mapping study, for example, un-inoculated plant SLB disease scoring was only possible a week later than inoculated plant scoring, though the genetic architecture results were similar for each type of inoculation (Balint-Kurti et al., 2007) and the un-inoculated and inoculated plots were adjacent to each other. Transmission of SLB has also been shown to vary by plot configuration (Johnson and Haddad, 2011). Variation in space as well as in time increases the complexity of infection patterns and complicates experimental design. We therefore chose to focus our phyllosphere bacterial community comparisons on fungicide-treated vs. inoculated plots.

Beta diversity in this study was lower than the level for B73 measured in the Balint-Kurti et al. (2010) study; the two experiments varied in time of sampling, with our sampling occurring earlier in the season, and in the leaf sampling protocol, with the 2010 study samples taken with Triton-containing leaf washes as compared to paper filters in our study. We saw a decrease in beta diversity within pathogen-inoculated plots (Table 2), supporting the Balint-Kurti et al. (2011) model at that spatial scale. We did not observe consistent, large-effect beta diversity differences in the leaf bacterial community for the fertilizer stress treatment, which may be due to short-distance dispersion and resulting spatial scaling, as was seen in a recent study of leaf colonization over time (Maignien et al., 2014). In previous studies of SLB (Carson et al., 2004), the inbred B73 was shown to have lower resistance to SLB than the inbred Mo17. In a mapping experiment using a population derived from a B73 × Mo17 cross, for most of the quantitative trait loci identified B73 alleles were associated with fewer taxa and more beta diversity than Mo17 alleles (Balint-Kurti et al., 2010). In the present study, diversity and disease severity were measured in the same experiment, allowing for a more controlled evaluation of this relationship within the B73 genotype. The number of bacterial taxa detected was similar to the number detected with non-nested ARISA in the Balint-Kurti et al. (2010) study, though in our current study taxa numbers varied over a wider range. Bacterial epiphyte diversity would be predicted by the Balint-Kurti et al. (2011) model to decrease under fertilized, pathogen-inoculated conditions; we did observe low ribotype diversity in pathogen-inoculated conditions, supporting that aspect of the model.

We observed a striking decline in the importance of specific ribotypes contributing substantially to bacterial community differences under conditions favoring severe pathogen effect (Figure 2B), suggesting that beneficial ribotypes may no longer be at a selective advantage when pathogen load is high. Enrichment of specific ribotypes was seen in field experiments with lettuce inoculated with human pathogenic bacteria (Williams et al., 2013). The consistent presence of specific suppressive bacterial ribotypes on the leaf surface might allow for the fungal pathogen to be outcompeted for space and resources, leading to changes in allocation of resources and thus increased allocation of resources to plant reproductive tissues (cob and seed). When pathogen loads are heavy, these beneficial communities might decline. Antagonists of pathogens can be easily identified but often show inconsistent effects across trials (Hadar and Papadopoulou, 2012). This may be due to specific interactions with abiotic factors such as temperature and humidity (De Curtis et al., 2012), or fertilizer as in our study, as well as the range of options for the composition of an optimal bacterial community for protection of plant health.

Our results suggest that specific phyllosphere microbial community sets could be evaluated as a potential early season breeding target for increase in yield overall in areas where pathogen pressure is high. A PCR-based or sequence-count-based test of bacterial presence or diversity using leaf samples already taken for genotyping may be suitable as a component of a screen for plants that perform better in a disease-enhancing environment, as these data can be collected before end-of-season disease severity scores are available. Further testing in production hybrids and across typical breeding environments and modeling of potential early-season assay value is suggested, along with further testing for bacterial species present within the seed to determine if prediction before planting is possible.

### Conflict of interest statement

The authors declare that the research was conducted in the absence of any commercial or financial relationships that could be construed as a potential conflict of interest.

## Supplementary material

Supplemental File 1 **Supplemental Methods and Results and Tables S1–S6**.

Supplemental File 2 **Plant trait data**.

Supplemental File 3 **Microbial epiphyte ribotype abundance data**.

Supplemental File 4 **Ribotype abundance file S3 sample key**.

Supplemental File 5 **SIMPER bacterial OTU contributions to community differences**.
