# Supplementary material for: Southern leaf blight disease severity is correlated with decreased maize leaf epiphytic bacterial species richness and the phyllosphere bacterial diversity decline is enhanced by nitrogen fertilization
Source: Front Plant Sci. 2014 Aug 15;5:403. doi: 10.3389/fpls.2014.00403 (PMC4133650; doi:10.3389/fpls.2014.00403)
Supplement: Supplemental File 1 — Supplemental Methods and Results and Tables S1–S6. [file DataSheet1.DOC]

**Supplemental File S1 Supplemental Results**

Map of Experimental Field Plots

|  | inoculated with SLB pathogen | | | | | | | | | | | | fungicide-treated | | | | | | | | | | | | |  |
| --- | --- | --- | --- | --- | --- | --- | --- | --- | --- | --- | --- | --- | --- | --- | --- | --- | --- | --- | --- | --- | --- | --- | --- | --- | --- | --- |
| fertilized |  |  |  | B73 |  |  |  |  |  |  |  |  | |  |  |  | B73 |  |  |  |  |  |  |  |  | |
|  |  |  |  |  |  |  |  |  | B73 |  | B73 |  | |  |  |  |  |  |  |  |  | B73 |  | B73 |  | |
|  | B73 |  |  |  |  |  |  | B73 |  |  |  |  | | B73 |  |  |  |  |  |  | B73 |  |  |  |  | |
|  |  | B73 |  |  | B73 |  |  |  |  | B73 |  |  | |  | B73 |  |  | B73 |  |  |  |  | B73 |  |  | |
|  |  |  |  |  |  | B73 |  |  |  |  |  |  | |  |  |  |  |  | B73 |  |  |  |  |  |  | |
|  |  |  |  |  |  |  |  |  |  |  |  | B73 | |  |  |  |  |  |  |  |  |  |  |  | B73 | |
| unfertilized |  |  |  | B73 |  |  |  |  |  |  |  |  | |  |  |  | B73 |  |  |  |  |  |  |  |  | |
|  |  |  |  |  |  |  |  |  | B73 |  | B73 |  | |  |  |  |  |  |  |  |  | B73 |  | B73 |  | |
|  | B73 |  |  |  |  |  |  | B73 |  |  |  |  | | B73 |  |  |  |  |  |  | B73 |  |  |  |  | |
|  |  | B73 |  |  | B73 |  |  |  |  | B73 |  |  | |  | B73 |  |  | B73 |  |  |  |  | B73 |  |  | |
|  |  |  |  |  |  | B73 |  |  |  |  |  |  | |  |  |  |  |  | B73 |  |  |  |  |  |  | |
|  |  |  |  |  |  |  |  |  |  |  |  | B73 | |  |  |  |  |  |  |  |  |  |  |  | B73 | |

Bacterial Diversity Statistical Analyses

The spread of the species distribution with each stress treatment type was analyzed using permdisp2; permanova was used to test for overall differences in location and/or distribution. Note that the Bray-Curtis distance measure has an inherent row-level standardization.

Supplemental Table S1 Robustness of Diversity Tests to Distribution and Method Parameters

| transformation | standardization | Distance measure | Permanova P(MonteCarlo) | Permdisp2 overall model uncorrected P(MonteCarlo) |
| --- | --- | --- | --- | --- |
| none | none | Bray-Curtis | Fertilizer=0.002  Pathogen=0.001 | P=0.177 |
| none | none | Euclidian | Pathogen=0.001 | P=0.03100§ |
| none | Z-score | Bray-Curtis | Pathogen=0.002 | P=0.002* |
| none | Z-score | Euclidian | Pathogen=0.001 | P=0.137 |
| Square-root | none | Bray-Curtis | Fertilizer=0.002  Pathogen=0.001 | P=0.221 |
| Square-root | none | Euclidian | Pathogen=0.001 | P=0.327 |
| Square-root | Z-score | Bray-Curtis | Pathogen=0.002 | P=0.808 |
| Square-root | Z-score | Euclidian | Pathogen=0.001 | P=0.108 |
| Fourth-root | none | Bray-Curtis | Fertilizer=0.002  Pathogen=0.001 | P=0.209 |
| Fourth-root | none | Euclidian | Pathogen=0.001 | P=0.144 |
| Fourth-root | Z-score | Bray-Curtis | Pathogen=0.001 | P=0.001* |
| Fourth-root | Z-score | Euclidian | Pathogen=0.001 | P=0.109 |
| Presence-absence | none | Bray-Curtis | Fertilizer=0.003  Pathogen=0.001 | P=0.195 |
| Presence-absence | none | Euclidian | Pathogen=0.001 | P=0.239 |
| Presence-absence | Z-score | Bray-Curtis | Pathogen=0.001 | P=0.002* |
| Presence-absence | Z-score | Euclidian | Pathogen=0.001 | P=0.002* |

*permutation error generated

§mean distance from centroid for unfertilized fungicide=47.12 , for unfertilized pathogen-inoculated=30.81, pairwise P=0.007 (significant after multiple-test correction).

Supplemental Table S2 Distribution Analysis of Chaol Alpha Diversity

|  |  | Chao1 Best-fit Distribution |
| --- | --- | --- |
| Pathogen-inoculated | fertilized | Weibull |
| Pathogen-inoculated | unfertilized | Weibull |
| Fungicide | fertilized | Mixture Normal |
| Fungicide | unfertilized | Normal |

Supplemental Table S3 Median Abundance Values of Top Eleven OTU from SIMPER Analysis

| **SLB** | | **No** | | **Yes** | |
| --- | --- | --- | --- | --- | --- |
| **Nitrogen** |  | **No** | **Yes** | **No** | **Yes** |
| OTU200 |  | 257.5 | 0 | 0 | 0 |
| OTU266 |  | 311 | 372 | 481.5 | 0 |
| OTU268 |  | 2332 | 1968.5 | 0 | 0 |
| OTU269 |  | 851 | 314 | 33 | 0 |
| OTU274 |  | 0 | 74.5 | 0 | 0 |
| OTU348 |  | 244.5 | 125 | 0 | 0 |
| OTU465 |  | 0 | 99.5 | 0 | 0 |
| OTU467 |  | 0 | 0 | 52 | 69.5 |
| OTU474 |  | 0 | 0 | 103.5 | 0 |
| OTU493 |  | 0 | 0 | 427.5 | 0 |
| OTU496 |  | 0 | 0 | 280 | 0 |

Statistical Analysis of Plant Traits

SAS JMP v11Pro was used to analyze differences in all trait measurements using a factorial model. The model was fit to each trait separately. Least squared means were compared among all treatment groups and Tukey-Kramer’s post-hoc test was implemented to determine which differences were significant (Supplemental Table 1). All interactions were included in the model. A multivariate manova was conducted on all plant traits together (Supplemental Table 2). Since disease severity rating is from a rating scale, we also tested significance of this trait using logistic regression (Supplemental Table 3). The variable names were shortened for statistical analysis and the key is provided below. Graphical details on post-hoc tests are shown for significant model factors, with significant pairwise comparisons in red type.

Variable name key:

Pathogen-inoculated = SLB level Yes

Fungicide = SLB level No

Fertilized = Nitrogen level yes

Unfertilized = Nitrogen level no

**Supplemental Table 4 Statistical Tests Results for Plant Traits**

***Response Height_in***

Whole Model

Summary of Fit

| RSquare | 0.495698 |
| --- | --- |
| RSquare Adj | 0.485874 |
| Root Mean Square Error | 10.25124 |
| Mean of Response | 62.62658 |
| Observations (or Sum Wgts) | 158 |

**Analysis of Variance**

| **Source** | **DF** | **Sum of Squares** | **Mean Square** | **F Ratio** |
| --- | --- | --- | --- | --- |
| Model | 3 | 15907.423 | 5302.47 | 50.4575 |
| Error | 154 | 16183.545 | 105.09 | **Prob > F** |
| C. Total | 157 | 32090.968 |  | <.0001 |

**Parameter Estimates**

| **Term** |  | **Estimate** | **Std Error** | **t Ratio** | **Prob>|t|** |
| --- | --- | --- | --- | --- | --- |
| Intercept |  | 62.613141 | 0.81561 | 76.77 | <.0001 |
| Nitrogen[no] |  | -8.925 | 0.81561 | -10.94 | <.0001 |
| SLB[No] |  | -1.061859 | 0.81561 | -1.30 | 0.1949 |
| Nitrogen[no]*SLB[No] |  | -4.575 | 0.81561 | -5.61 | <.0001 |

**Effect Tests**

| **Source** | **Nparm** | **DF** | **Sum of Squares** | **F Ratio** | **Prob > F** |  |
| --- | --- | --- | --- | --- | --- | --- |
| Nitrogen | 1 | 1 | 12583.572 | 119.7432 | <.0001 |  |
| SLB | 1 | 1 | 178.123 | 1.6950 | 0.1949 |  |
| Nitrogen*SLB | 1 | 1 | 3306.509 | 31.4642 | <.0001 |  |

**Nitrogen**

**Least Squares Means Table**

| **Level** | **Least Sq Mean** |  | **Std Error** | **Mean** |
| --- | --- | --- | --- | --- |
| no | 53.688141 |  | 1.1534472 | 53.7595 |
| yes | 71.538141 |  | 1.1534472 | 71.4937 |

**LSMeans Differences Student's t**

α=0.050 t=1.97549

LSMean[i] By LSMean[j]

| Mean[i]-Mean[j]  Std Err Dif  Lower CL Dif  Upper CL Dif | no | yes |
| --- | --- | --- |
| no | 0  0  0  0 | -17.85  1.63122  -21.072  -14.628 |
| yes | 17.85  1.63122  14.6275  21.0725 | 0  0  0  0 |

| **Level** |  |  | **Least Sq Mean** |
| --- | --- | --- | --- |
| yes | A |  | 71.538141 |
| no |  | B | 53.688141 |

Levels not connected by same letter are significantly different.

**SLB**

**Least Squares Means Table**

| **Level** | **Least Sq Mean** |  | **Std Error** | **Mean** |
| --- | --- | --- | --- | --- |
| No | 61.551282 |  | 1.1607245 | 61.5513 |
| Yes | 63.675000 |  | 1.1461237 | 63.6750 |

**Nitrogen*SLB**

**Least Squares Means Table**

| **Level** | **Least Sq Mean** |  | **Std Error** |
| --- | --- | --- | --- |
| no,No | 48.051282 |  | 1.6415124 |
| no,Yes | 59.325000 |  | 1.6208636 |
| yes,No | 75.051282 |  | 1.6415124 |
| yes,Yes | 68.025000 |  | 1.6208636 |

**LSMeans Differences Tukey HSD**

α=0.050 Q=2.59731

LSMean[i] By LSMean[j]

| Mean[i]-Mean[j]  Std Err Dif  Lower CL Dif  Upper CL Dif | no,No | no,Yes | yes,No | yes,Yes |
| --- | --- | --- | --- | --- |
| no,No | 0  0  0  0 | -11.274  2.30689  -17.265  -5.282 | -27  2.32145  -33.03  -20.97 | -19.974  2.30689  -25.965  -13.982 |
| no,Yes | 11.2737  2.30689  5.28199  17.2654 | 0  0  0  0 | -15.726  2.30689  -21.718  -9.7346 | -8.7  2.29225  -14.654  -2.7463 |
| yes,No | 27  2.32145  20.9705  33.0295 | 15.7263  2.30689  9.73455  21.718 | 0  0  0  0 | 7.02628  2.30689  1.03455  13.018 |
| yes,Yes | 19.9737  2.30689  13.982  25.9654 | 8.7  2.29225  2.74631  14.6537 | -7.0263  2.30689  -13.018  -1.0346 | 0  0  0  0 |

| **Level** |  |  |  |  | **Least Sq Mean** |
| --- | --- | --- | --- | --- | --- |
| yes,No | A |  |  |  | 75.051282 |
| yes,Yes |  | B |  |  | 68.025000 |
| no,Yes |  |  | C |  | 59.325000 |
| no,No |  |  |  | D | 48.051282 |

Levels not connected by same letter are significantly different.

***Response seed_wt***

**Whole Model**

Summary of Fit

| RSquare | 0.1699 |
| --- | --- |
| RSquare Adj | 0.144226 |
| Root Mean Square Error | 0.601035 |
| Mean of Response | 5.568614 |
| Observations (or Sum Wgts) | 101 |

**Analysis of Variance**

| **Source** | **DF** | **Sum of Squares** | **Mean Square** | **F Ratio** |
| --- | --- | --- | --- | --- |
| Model | 3 | 7.171870 | 2.39062 | 6.6178 |
| Error | 97 | 35.040536 | 0.36124 | **Prob > F** |
| C. Total | 100 | 42.212406 |  | 0.0004 |

**Parameter Estimates**

| **Term** |  | **Estimate** | **Std Error** | **t Ratio** | **Prob>|t|** |
| --- | --- | --- | --- | --- | --- |
| Intercept |  | 5.5153961 | 0.06507 | 84.76 | <.0001 |
| Nitrogen[no] |  | -0.100011 | 0.06507 | -1.54 | 0.1276 |
| SLB[No] |  | 0.0161314 | 0.06507 | 0.25 | 0.8047 |
| Nitrogen[no]*SLB[No] |  | -0.250747 | 0.06507 | -3.85 | 0.0002 |

**Effect Tests**

| **Source** | **Nparm** | **DF** | **Sum of Squares** | **F Ratio** | **Prob > F** |  |
| --- | --- | --- | --- | --- | --- | --- |
| Nitrogen | 1 | 1 | 0.8533569 | 2.3623 | 0.1276 |  |
| SLB | 1 | 1 | 0.0222011 | 0.0615 | 0.8047 |  |
| Nitrogen*SLB | 1 | 1 | 5.3641602 | 14.8492 | 0.0002 |  |

**Nitrogen**

**Least Squares Means Table**

| **Level** | **Least Sq Mean** |  | **Std Error** | **Mean** |
| --- | --- | --- | --- | --- |
| no | 5.4153846 |  | 0.10816731 | 5.45938 |
| yes | 5.6154076 |  | 0.07236367 | 5.61928 |

**SLB**

**Least Squares Means Table**

| **Level** | **Least Sq Mean** |  | **Std Error** | **Mean** |
| --- | --- | --- | --- | --- |
| No | 5.5315275 |  | 0.09760778 | 5.69229 |
| Yes | 5.4992647 |  | 0.08607780 | 5.45660 |
|  |  |  |  |  |

**Nitrogen*SLB**

**Least Squares Means Table**

| **Level** | **Least Sq Mean** |  | **Std Error** |
| --- | --- | --- | --- |
| no,No | 5.1807692 |  | 0.16669702 |
| no,Yes | 5.6500000 |  | 0.13788680 |
| yes,No | 5.8822857 |  | 0.10159340 |
| yes,Yes | 5.3485294 |  | 0.10307659 |

**LSMeans Differences Tukey HSD**

α=0.050 Q=2.61413

LSMean[i] By LSMean[j]

| Mean[i]-Mean[j]  Std Err Dif  Lower CL Dif  Upper CL Dif | no,No | no,Yes | yes,No | yes,Yes |
| --- | --- | --- | --- | --- |
| no,No | 0  0  0  0 | -0.4692  0.21633  -1.0348  0.0963 | -0.7015  0.19522  -1.2118  -0.1912 | -0.1678  0.19599  -0.6801  0.34459 |
| no,Yes | 0.46923  0.21633  -0.0963  1.03476 | 0  0  0  0 | -0.2323  0.17127  -0.68  0.21544 | 0.30147  0.17216  -0.1486  0.75151 |
| yes,No | 0.70152  0.19522  0.1912  1.21184 | 0.23229  0.17127  -0.2154  0.68001 | 0  0  0  0 | 0.53376  0.14473  0.15542  0.91209 |
| yes,Yes | 0.16776  0.19599  -0.3446  0.68011 | -0.3015  0.17216  -0.7515  0.14857 | -0.5338  0.14473  -0.9121  -0.1554 | 0  0  0  0 |

| **Level** |  |  | **Least Sq Mean** |
| --- | --- | --- | --- |
| yes,No | A |  | 5.8822857 |
| no,Yes | A | B | 5.6500000 |
| yes,Yes |  | B | 5.3485294 |
| no,No |  | B | 5.1807692 |

Levels not connected by same letter are significantly different.

***Response diameter***

**Whole Model**

Summary of Fit

| RSquare | 0.306094 |
| --- | --- |
| RSquare Adj | 0.289031 |
| Root Mean Square Error | 1.496297 |
| Mean of Response | 11.94397 |
| Observations (or Sum Wgts) | 126 |

**Analysis of Variance**

| **Source** | **DF** | **Sum of Squares** | **Mean Square** | **F Ratio** |
| --- | --- | --- | --- | --- |
| Model | 3 | 120.48978 | 40.1633 | 17.9388 |
| Error | 122 | 273.14643 | 2.2389 | **Prob > F** |
| C. Total | 125 | 393.63622 |  | <.0001 |

**Parameter Estimates**

| **Term** |  | **Estimate** | **Std Error** | **t Ratio** | **Prob>|t|** |
| --- | --- | --- | --- | --- | --- |
| Intercept |  | 11.739054 | 0.14236 | 82.46 | <.0001 |
| Nitrogen[no] |  | -0.953439 | 0.14236 | -6.70 | <.0001 |
| SLB[No] |  | 0.2045014 | 0.14236 | 1.44 | 0.1534 |
| Nitrogen[no]*SLB[No] |  | -0.229492 | 0.14236 | -1.61 | 0.1095 |

**Effect Tests**

| **Source** | **Nparm** | **DF** | **Sum of Squares** | **F Ratio** | **Prob > F** |  |
| --- | --- | --- | --- | --- | --- | --- |
| Nitrogen | 1 | 1 | 100.42619 | 44.8550 | <.0001 |  |
| SLB | 1 | 1 | 4.62012 | 2.0636 | 0.1534 |  |
| Nitrogen*SLB | 1 | 1 | 5.81830 | 2.5987 | 0.1095 |  |

**Nitrogen**

**Least Squares Means Table**

| **Level** | **Least Sq Mean** |  | **Std Error** | **Mean** |
| --- | --- | --- | --- | --- |
| no | 10.785616 |  | 0.22791292 | 10.7943 |
| yes | 12.692493 |  | 0.17064846 | 12.6756 |

**LSMeans Differences Student's t**

α=0.050 t=1.9796

LSMean[i] By LSMean[j]

| Mean[i]-Mean[j]  Std Err Dif  Lower CL Dif  Upper CL Dif | no | yes |
| --- | --- | --- |
| no | 0  0  0  0 | -1.9069  0.28472  -2.4705  -1.3432 |
| yes | 1.90688  0.28472  1.34325  2.47051 | 0  0  0  0 |

| **Level** |  |  | **Least Sq Mean** |
| --- | --- | --- | --- |
| yes | A |  | 12.692493 |
| no |  | B | 10.785616 |

Levels not connected by same letter are significantly different.

**SLB**

**Least Squares Means Table**

| **Level** | **Least Sq Mean** |  | **Std Error** | **Mean** |
| --- | --- | --- | --- | --- |
| No | 11.943556 |  | 0.22385404 | 12.4123 |
| Yes | 11.534553 |  | 0.17593908 | 11.6040 |

**Nitrogen*SLB**

**Least Squares Means Table**

| **Level** | **Least Sq Mean** |  | **Std Error** |
| --- | --- | --- | --- |
| no,No | 10.760625 |  | 0.37407429 |
| no,Yes | 10.810606 |  | 0.26047190 |
| yes,No | 13.126486 |  | 0.24598974 |
| yes,Yes | 12.258500 |  | 0.23658535 |

***Response Score***

**Whole Model**

Summary of Fit

| RSquare | 0.774603 |
| --- | --- |
| RSquare Adj | 0.750454 |
| Root Mean Square Error | 0.519056 |
| Mean of Response | 6.53125 |
| Observations (or Sum Wgts) | 32 |

**Analysis of Variance**

| **Source** | **DF** | **Sum of Squares** | **Mean Square** | **F Ratio** |
| --- | --- | --- | --- | --- |
| Model | 3 | 25.925000 | 8.64167 | 32.0751 |
| Error | 28 | 7.543750 | 0.26942 | **Prob > F** |
| C. Total | 31 | 33.468750 |  | <.0001 |

**Parameter Estimates**

| **Term** |  | **Estimate** | **Std Error** | **t Ratio** | **Prob>|t|** |
| --- | --- | --- | --- | --- | --- |
| Intercept |  | 6.728125 | 0.098399 | 68.38 | <.0001 |
| Nitrogen[no] |  | 0.303125 | 0.098399 | 3.08 | 0.0046 |
| SLB[No] |  | 0.921875 | 0.098399 | 9.37 | <.0001 |
| Nitrogen[no]*SLB[No] |  | 0.046875 | 0.098399 | 0.48 | 0.6375 |

**Effect Tests**

| **Source** | **Nparm** | **DF** | **Sum of Squares** | **F Ratio** | **Prob > F** |  |
| --- | --- | --- | --- | --- | --- | --- |
| Nitrogen | 1 | 1 | 2.556793 | 9.4900 | 0.0046 |  |
| SLB | 1 | 1 | 23.648098 | 87.7742 | <.0001 |  |
| Nitrogen*SLB | 1 | 1 | 0.061141 | 0.2269 | 0.6375 |  |

**Nitrogen**

**Least Squares Means Table**

| **Level** | **Least Sq Mean** |  | **Std Error** | **Mean** |
| --- | --- | --- | --- | --- |
| no | 7.0312500 |  | 0.15892794 | 6.70833 |
| yes | 6.4250000 |  | 0.11606456 | 6.42500 |

**LSMeans Differences Student's t**

α=0.050 t=2.04841

LSMean[i] By LSMean[j]

| Mean[i]-Mean[j]  Std Err Dif  Lower CL Dif  Upper CL Dif | no | yes |
| --- | --- | --- |
| no | 0  0  0  0 | 0.60625  0.1968  0.20313  1.00937 |
| yes | -0.6062  0.1968  -1.0094  -0.2031 | 0  0  0  0 |

| **Level** |  |  | **Least Sq Mean** |
| --- | --- | --- | --- |
| no | A |  | 7.0312500 |
| yes |  | B | 6.4250000 |

Levels not connected by same letter are significantly different.

**SLB**

**Least Squares Means Table**

| **Level** | **Least Sq Mean** |  | **Std Error** | **Mean** |
| --- | --- | --- | --- | --- |
| No | 7.6500000 |  | 0.15353898 | 7.50000 |
| Yes | 5.8062500 |  | 0.12310506 | 5.77778 |

**LSMeans Differences Student's t**

α=0.050 t=2.04841

LSMean[i] By LSMean[j]

| Mean[i]-Mean[j]  Std Err Dif  Lower CL Dif  Upper CL Dif | No | Yes |
| --- | --- | --- |
| No | 0  0  0  0 | 1.84375  0.1968  1.44063  2.24687 |
| Yes | -1.8438  0.1968  -2.2469  -1.4406 | 0  0  0  0 |

| **Level** |  |  | **Least Sq Mean** |
| --- | --- | --- | --- |
| No | A |  | 7.6500000 |
| Yes |  | B | 5.8062500 |

Levels not connected by same letter are significantly different.

**Nitrogen*SLB**

**Least Squares Means Table**

| **Level** | **Least Sq Mean** |  | **Std Error** |
| --- | --- | --- | --- |
| no,No | 8.0000000 |  | 0.25952825 |
| no,Yes | 6.0625000 |  | 0.18351418 |
| yes,No | 7.3000000 |  | 0.16414008 |
| yes,Yes | 5.5500000 |  | 0.16414008 |

**Supplemental Table S5 MANOVA Results for Plant Traits**

**Response Specification**

To construct the linear combinations across responses,

|  |  |
| --- | --- |
| N | 28 |
| DFE | 24 |

**Parameter Estimates**

|  | **Height_in** | **seed_wt** | **diameter** | **Score** |
| --- | --- | --- | --- | --- |
| Intercept | 67.4833333 | 5.57984722 | 12.3232222 | 6.71805556 |
| Nitrogen[no] | -3.9333333 | 0.01240278 | -0.9952222 | 0.33194444 |
| SLB[No] | 1.93333333 | 0.00084722 | 0.08622222 | 0.89305556 |
| Nitrogen[no]*SLB[No] | -2.9833333 | -0.1905972 | -0.3042222 | 0.05694444 |

**Least Squares Means**

**Overall Means**

| *Overall Means* **Height_in** | **seed_wt** | **diameter** | **Score** |
| --- | --- | --- | --- |
| 68.75 | 5.57535714 | 12.6725 | 6.53571429 |

**Nitrogen**

| Nitrogen | **Height_in** | **seed_wt** | **diameter** | **Score** |
| --- | --- | --- | --- | --- |
| no | 63.55 | 5.59225 | 11.328 | 7.05 |
| yes | 71.4166667 | 5.56744444 | 13.3184444 | 6.38611111 |

**SLB**

| SLB | **Height_in** | **seed_wt** | **diameter** | **Score** |
| --- | --- | --- | --- | --- |
| No | 69.4166667 | 5.58069444 | 12.4094444 | 7.61111111 |
| Yes | 65.55 | 5.579 | 12.237 | 5.825 |

Nitrogen*SLB

| Nitrogen*SLB | **Height_in** | **seed_wt** | **diameter** | **Score** |
| --- | --- | --- | --- | --- |
| no,No | 62.5 | 5.4025 | 11.11 | 8 |
| no,Yes | 64.6 | 5.782 | 11.546 | 6.1 |
| yes,No | 76.3333333 | 5.75888889 | 13.7088889 | 7.22222222 |
| yes,Yes | 66.5 | 5.376 | 12.928 | 5.55 |

**Identity**

**Whole Model**

| **Test** | **Value** | **Approx. F** | **NumDF** | **DenDF** | **Prob>F** |
| --- | --- | --- | --- | --- | --- |
| Wilks' Lambda | 0.0980301 | 6.5423 | 12 | 55.852 | <.0001 |
| Pillai's Trace | 1.3633153 | 4.7896 | 12 | 69 | <.0001 |
| Hotelling-Lawley | 4.8438405 | 7.9385 | 12 | 59 | <.0001 |
| Roy's Max Root | 3.8180101 | 21.9536 | 4 | 23 | <.0001 |

**Intercept**

| **Test** | **Value** | **Exact F** | **NumDF** | **DenDF** | **Prob>F** |
| --- | --- | --- | --- | --- | --- |
| F Test | 407.62583 | 2140.0356 | 4 | 21 | <.0001 |

**Nitrogen**

| **Test** | **Value** | **Exact F** | **NumDF** | **DenDF** | **Prob>F** |
| --- | --- | --- | --- | --- | --- |
| F Test | 0.9981462 | 5.2403 | 4 | 21 | 0.0044 |

**SLB**

| **Test** | **Value** | **Exact F** | **NumDF** | **DenDF** | **Prob>F** |
| --- | --- | --- | --- | --- | --- |
| F Test | 3.1528383 | 16.5524 | 4 | 21 | <.0001 |

**Nitrogen*SLB**

| **Test** | **Value** | **Exact F** | **NumDF** | **DenDF** | **Prob>F** |
| --- | --- | --- | --- | --- | --- |
| F Test | 0.2047905 | 1.0752 | 4 | 21 | 0.3938 |

**Supplemental Table S6 Ordinal Logistic Fit for Score Trait**

**Whole Model Test**

| **Model** | **-LogLikelihood** | **DF** | **ChiSquare** | **Prob>ChiSq** |
| --- | --- | --- | --- | --- |
| Difference | 22.818424 | 3 | 45.63685 | <.0001 |
| Full | 32.560073 |  |  |  |
| Reduced | 55.378497 |  |  |  |

| RSquare (U) | 0.4120 |
| --- | --- |
| Observations (or Sum Wgts) | 32 |

Converged by Objective

**Effect Likelihood Ratio Tests**

| **Source** | **Nparm** | **DF** | **L-R ChiSquare** | **Prob>ChiSq** |  |
| --- | --- | --- | --- | --- | --- |
| Nitrogen | 1 | 1 | 11.0523083 | 0.0009 |  |
| SLB | 1 | 1 | 42.0362405 | <.0001 |  |
| Nitrogen*SLB | 1 | 1 | 1.13417741 | 0.2869 |  |
